# Editorial: Biological drivers of vector–pathogen interactions – vol II

**DOI:** 10.3389/fcimb.2023.1170834

**Published:** 2023-03-06

**Authors:** Ryan O. M. Rego, Job E. Lopez, Alejandro Cabesas-Cruz

**Affiliations:** ^1^ Biology Centre, Institute of Parasitology, Czech Academy of Sciences, České Budějovice, Czechia; ^2^ Faculty of Science, University of South Bohemia, České Budějovice, Czechia; ^3^ Department of Molecular Virology and Microbiology, Baylor College of Medicine, Houston, TX, United States; ^4^ Department of Pediatrics, National School of Tropical Medicine, Baylor College of Medicine, Houston, TX, United States; ^5^ UMR BIPAR, INRAE, ANSES, Ecole Nationale Vétérinaire d’Alfort, Université Paris-Est, Maisons-Alfort, France

**Keywords:** vector, pathogen, saliva, surveillance, tick microbe interactions, tick microbiome, mosquito, dengue

Our quest as researchers to understand and push back on the spread of vector-borne diseases has improved significantly over the last few years. This is largely due to the contributions of ‘omics’ technologies which speed up research carried out and analysed on the vector, pathogen and the vector microbiome simultaneously. The faster we can work with the data obtained, the closer we are to our goals of attaining methods to protect animals and humans against the diseases and hopefully eradicate them in the not-so-distant future. To this end the contributions within this Research Topic are exactly what we need.

## Pathogen/vector surveillance

Constant surveillance studies are important, so as to be prepared for possible outbreaks and to control vector-borne diseases. Liu et al., collected 140,000 mosquitoes, belonging to various species from 2018-2020 in both rural and urban habitats. The authors show that one of the fully sequenced virus isolates they obtained from their collection in Zheigang, China was evolutionary related to the isolate originating from Cambodia, a distance of 3,500km. Using genomic data, the authors reported that the Dengue virus, which infected the local *Aedes albopictus* mosquitoes, led to the local Dengue Fever virus outbreak in this region in 2019 and displays a correlation with imported Dengue Fever cases from Cambodia at this time. Overall, the importance of such studies cannot be stressed enough. Sampling, whole genome sequencing, phylogenetic analysis, and the analysis of medical records are needed to build a complete story for local and national vector-borne disease surveillance.

## Microbiome

The review of Hodosi et al. focuses on the microbiome of the European castor bean tick, *Ixodes ricinus*. The microbiome present in the midgut, ovaries, and the salivary glands was evaluated. This review, in our opinion, appears to be the most complete until now. It details non-pathogenic (endosymbiotic and commensal bacteria) and pathogenic (viruses, bacteria, apicomplexans) colonizers of *I. ricinus*. The authors highlight interactions between the various bacteria in the microbiome. They also hypothesise the possibility that the tick could support the survival of *Wolbachia* species or the pathogen *Anaplasma phagocytophilum*. They suggest this because of literature showing that *Spiroplasma ixodetis* provides the tick with the complete metabolic pathway of folate biosynthesis necessary for cell division and DNA synthesis.

Adegoke et al. used a high-throughput sequencing approach to dissect how the blood meal as well as *Rickettsia parkeri* affected the bacterial complexity in tissues of the tick *Amblyomma maculatum*. They discerned the predominance of *Rickettsia* and *Francisella* species in the core microbiome of the tick. They also saw an increase in prevalence of *Candidatus midichloria* and Cutibacterium with *R. parkeri* infection. The authors used network analysis to look at microbial interactions in various tissues of the ticks. The showed that the tick genome across ontogeny was relatively stable and that there was a reduction in community diversity toward later developmental stages. Most importantly they showed that the interactions between the various microbial species are tissue-specific. This work will support other systems to study ticks/microbiome/pathogens interactions.

Couret et al. have provided a review, on what is the central theme of this Research topic and that is the various drivers of vector-pathogen interactions including temperature, humidity, environmental bacteria from the surroundings of the ticks and the prevalence of infection within these vectors. What is unique is that the authors focus on the North American *Ixodes scapularis* tick and hence cover literature not covered in the other review by Hodosi et al., which pertained to the European Ixodes tick. The authors have made a concrete effort to try and point readers to well recognized studies and reviews highlighting microbial diversity in *I. scapularis*.

## Tick hybrids as vectors of pathogens

Mating of different species within the same tick genus is relevant to disease ecology, as hybrid offspring could vector pathogens transmitted by both parental lineages. Until now this work has been done in the field of mosquito research. Here, Belova et al., worked at understanding the TBEV transmission dynamics by *I. ricinus*, *I. persulcatus* and hybrid nymphs. They did this by using two different TBEV subtypes. The one significant conclusion was that the hybrid ticks displayed the highest acquisition effectiveness and RNA copy numbers with one of the virus subtypes, but overall, the infection rate was not different between the hybrid and the two parental tick species.

## Modulation of vector saliva

We know that pathogen infection leads to transcriptional and proteome modulation in both ticks and mosquitoes. This work has been done by dissecting the salivary gland organs. In ticks, saliva has been obtained using chemical agents like pilocarpine. Filatov et al. provide the first proof-of-principle non-invasive method to collect saliva from *Ornithodoros moubata* soft ticks using an artificial membrane feeding system. They then analyzed secreted salivary proteins of *Borrelia duttonii*-infected and uninfected ticks using mass spectrometry analysis. Their results show distinct differences between infected versus uninfected ticks. This method paves the way for future work in understanding modulation of vector gene expression due to infection.

We would like to express our thanks to all authors who contributed towards this Research Topic and sharing more insight into the interactions at the level of the vector-pathogen.

## Author contributions

All authors listed have made a substantial, direct and intellectual contribution to the work, and approved it for publication.

